# P-1264. The Long View: Tracking Gram-negative Susceptibility Trends from 2010-2024 to Inform Stewardship

**DOI:** 10.1093/ofid/ofaf695.1455

**Published:** 2026-01-11

**Authors:** David S Burgess, Justin Clark, Katie B Olney, Donna R Burgess

**Affiliations:** University of Kentucky, Lexington, KY; University of Kentucky HealthCare, Lexington, Kentucky; University of Kentucky HealthCare, Lexington, Kentucky; UK HealthCare, Lexington, KY

## Abstract

**Background:**

The rise in antimicrobial resistance (AMR) among Gram-negative bacteria poses significant treatment challenges. This study evaluated susceptibility trends over a 15-year period for key Gram-negative organisms at UK HealthCare (UKHC) to guide stewardship strategies and empirical therapy.

**Methods:**

A retrospective review of cumulative antimicrobial susceptibility data from 2010 to 2024 was conducted for *K. aerogenes*, *E. coli*, *K. pneumoniae*, *P. aeruginosa*, *S. marcescens*, and *E. cloacae*. Susceptibility to cefepime, piperacillin-tazobactam, levofloxacin, ceftriaxone, trimethoprim-sulfamethoxazole (TMP-SMX), and meropenem were analyzed. Trend lines were generated using linear regression to assess changes over time.

**Results:**

Over the 15-year period, notable declines in susceptibility were observed among *Enterobacterales*, particularly *K. pneumoniae* and *E. coli*. Cefepime susceptibility declined from 98% to 81% (R² = 0.8291) in *K. pneumoniae* and from 95% to 83% (R² = 0.7809) in *E. coli*. For *K. pneumoniae*, susceptibilities fell for piperacillin-tazobactam (90% to 74%, R² = 0.5163), ceftriaxone (91% to 81%, R² = 0.7448) and TMP-SMX (95% to 80%, R² = 0.6504). *E. coli* also showed declining susceptibility to ceftriaxone (R² = 0.4627) and cefepime (R² = 0.7809) though piperacillin-tazobactam susceptibility remained stable above 90%. *K. aerogenes* exhibited declines across all agents, particularly levofloxacin and cefepime. In contrast, *P. aeruginosa* demonstrated stable or slightly improving susceptibility to cefepime, meropenem, and piperacillin-tazobactam. *S. marcescens* showed minimal variability in susceptibility, while *E. cloacae* experienced modest declines, especially to ceftriaxone and piperacillin-tazobactam.

**Conclusion:**

At UKHC, antimicrobial resistance among *Enterobacterales*, especially *K. pneumoniae* and *E. coli*, continues to worsen, driven by declining susceptibility to key beta-lactams and fluoroquinolones. Meanwhile, susceptibility among *P. aeruginosa* and *S. marcescens* has remained relatively stable. These findings highlight the critical need for robust antimicrobial stewardship interventions and local, data-driven empiric prescribing, supported by ongoing surveillance and rapid diagnostics.

**Disclosures:**

All Authors: No reported disclosures

